# Prospects for liquid biopsy using microRNA and extracellular vesicles in breast cancer

**DOI:** 10.1007/s12282-024-01563-9

**Published:** 2024-03-30

**Authors:** Takahiro Ochiya, Kazuki Hashimoto, Akihiko Shimomura

**Affiliations:** 1 Department of Molecular and Cellular Medicine, Center for Future Medical Research, Institute of Medical Science, Tokyo Medical University, 6-7-1 Nishishinjuku, Shinjyuku-ku, Tokyo, 160-0023 Japan; 2 Department of Breast Surgery, National Center for Global Health and Medicine, 1-21-1 Toyama, Shinjuku-ku, Tokyo, 162-8655 Japan; 3 Department of Breast and Medical Oncology, Genetic Medicine, General Medical Oncology, National Center for Global Health and Medicine, 1-21-1 Toyama, Shinjuku-ku, Tokyo, 162-8655 Japan

**Keywords:** Liquid biopsy, Extracellular vesicles, MicroRNA

## Abstract

Among the analytes circulating in body fluids, microRNAs, a type of non-coding RNA and known to exist 2655 in primates, have attracted attention as a novel biomarker for cancer screening. MicroRNAs are signaling molecules with important gene expression regulatory functions that can simultaneously control many gene functions and multiple different pathways in living organisms. These microRNAs are transported in extracellular vesicles (EVs), which are lipid bilayers with 50–150 nm in diameter, and are used as communication tools between cells. Furthermore, the EVs that carry these microRNAs circulate in the bloodstream and have other important implications for understanding the pathogenesis and diagnosis of breast cancer. The greatest benefit from cancer screening is the reduction in breast cancer mortality rate through early detection. Other benefits include reduced incidence of breast cancer, improved quality of life, prognosis prediction, contribution to personalized medicine, and relative healthcare cost containment. This paper outlines the latest developments in liquid biopsy for breast cancer, especially focusing on microRNA and EV diagnostics.

## Introduction

The number of deaths due to cancer in Japan is the leading cause of death overall, and we are living in an era in which one in two people will be diagnosed with cancer during their lifetime. However, recent advances in diagnosis and treatment have made early detection and treatment possible for some cancers, giving cancer patients more hope. Although cancer screening is considered to be a sure-fire way to reduce cancer mortality based on these advances in medical technology, the current situation is that countermeasure-based screening is only available for a small number of cancers, such as stomach, colon, lung, and breast cancer, and Japan has a very low screening uptake rate for all cancer types among developed countries, according to cancer screening by the Japan Cancer Society and others. Especially due to the COVID infection situation, there has been a sharp decline in the number of people undergoing cancer screening, and there have been notable cases of cancer being detected at an advanced stage [1]. The greatest benefit from cancer screening is the reduction in cancer mortality achieved through early detection. Other benefits include reduced incidence of targeted cancers, improved quality of life, and lower relative health care costs. On the other hand, to be sure, there are instances in which cancer screening suffers serious disadvantages as well as these benefits. A common problem with any cancer screening is the problem of "false negatives" and "false positives," as well as "overdiagnosis ". These disadvantages arise from the insufficient reliability of the test markers and their poor performance in discriminating cancer from cancer and normal from normal, and there is a need for more accurate markers. Furthermore, even when cancer is diagnosed, it may be a benign disease or disappear spontaneously before developing into cancer, and there is always the risk of over-diagnosing it as cancer [2]. In addition, the development of a simple test method that can detect multiple cancers and diseases with a single blood sample, such as in mass screening, is expected to lead to an increase in the uptake rate of cancer screening.

## Concept of liquid biopsy

**Fig. 1:**
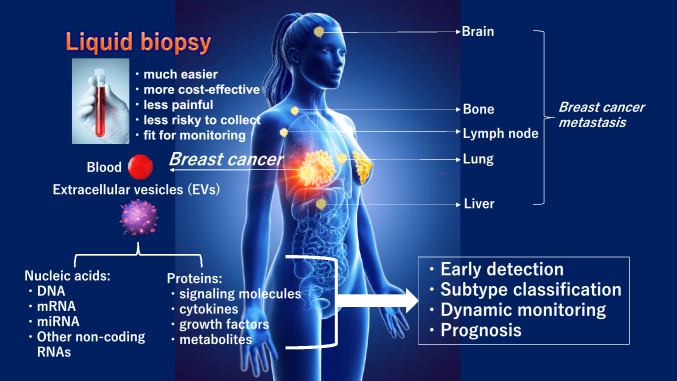
Novel Modalities of Breast Cancer Diagnosis. In contrast to solid biopsies, liquid biopsies are non-invasive and thus much easier, more cost-effective, less painful and less risky to collect. A small aliquot of blood as well as saliva and urine are enough to harvest extracellular vesicles (EVs) for detecting multimodal envoys, such as DNA, mRNA, microRNA, and other non-coding RNAs (nucleic acid-based) and several protein markers including EV surface proteins, EV-encapsulated proteins, and metabolites (protein-based). Those are expected for (1) early detection of breast cancer (cancer screening test) for identify resectable cancer; (2) subtype classification of breast cancer; (3) dynamic monitoring for evaluation of response to treatment; and (4) prognosis for timely detection of resectable metastatic breast cancer of brain, lung, lymph node and liver

To overcome these problems with current tumor markers, a new diagnostic method called "liquid biopsy" is attracting attention [3]. Liquid biopsy has multiple advantages, including low invasiveness, low patient burden, and reduced medical costs. Promising tools for liquid biopsy are (1) CTC (circulating tumor cells: "circulating tumor cells in the blood" or "circulating tumor cells in the peripheral blood"); (2) Cell-free DNA (DNA fragments that are thought to have been liberated from cancer cells present in the blood, especially fragments with genetic mutations that characterize cancer); (3) Cell-free microRNAs, so-called secreted microRNAs, are secreted not only from cancer cells but also from normal tissues and cells, and circulate in blood and other body fluids.; (4) Extracellular vesicles (EVs),; (5) Metabolites, since cancer cells use different metabolic pathways than normal cells, cancer-specific metabolites circulate in the blood of patients, etc. In this paper, we review secreted microRNAs and EVs that can detect cancer early with a single drop of blood, which has attracted the most attention among these metabolites (Fig. 1).

## Relationship between microRNAs and cancer

In an industry–government–academia collaboration supported by NEDO/AMED in Japanese agency, there is a project that fully utilized the biobanks of the National Cancer Center, Tokyo, Japan and the National Institute for Geriatrics and Gerontology, Aichi, Japan to conduct a comprehensive analysis of blood microRNAs in over 13 major cancers in the Japanese population, with a large-scale study for each cancer [4]. The results of these efforts are now in the process of being evaluated for clinical performance by three companies in Japan, with the cooperation of the Japan Cancer Society, and the first microRNA diagnosis for the early detection of cancer is about to be put into practical use. It is believed that microRNAs are suitable for early diagnosis because they are actively secreted by early cancer cells and surrounding cells, even when the cancer is still at a small stage, and help control the cancer microenvironment. Our human bodies have 2,655 types of microRNAs, and machine learning of the respective profiles in cancer and normal blood with the help of artificial intelligence and other tools has revealed that about 500 types of microRNAs are altered in cancer, and that the types and combinations of microRNAs in these alterations are different for each type of cancer [5]. Each microRNA plays a minute coordinating role in turning off and sometimes on the translation of proteins by regulating the mRNAs that its sequence recognizes, and an imbalance in the total expression of these microRNAs may be the cause of cancer, or it may be the cause of lifestyle-related diseases, chronic diseases, or dementia. It is also related to the onset of lifestyle-related diseases, chronic diseases, and dementia.

## Early detection of breast cancer by blood microRNAs

The aforementioned national project proved that it is possible to detect breast cancer in its early stages using microRNAs in combination of five different types. Dr. Akihiko Shimomura (now at the National Center for Global Health and Medicine) and colleagues involved in this study identified hundreds of microRNAs specifically found in the early stages of breast cancer by array analysis of microRNA profiles of stored sera from 1280 breast cancer patients, mainly stage I and II early-stage cancer, and from approximately 2000 healthy adults (age-matched, mostly women), Statistical processing using Fisher’s linear discriminant and combinatorial optimization identified five microRNA combinations that had the ability to discriminate breast cancer patients. The five microRNA combinations were identified as having the ability to distinguish breast cancer patients from healthy adults with an AUC = 0.971, which is a high level of discrimination between the two groups [6]. These microRNAs were able to distinguish breast cancer with a sensitivity of more than 90%, independent of breast cancer subtype or stage. However, such microRNAs, which were thought to be specific for breast cancer, have in some cases discriminated benign breast diseases from cancer (specificity is about 83%), and further improvement in accuracy is needed. It has been reported that microRNAs in blood contribute not only to the early detection of breast cancer, but also to the determination of lymph node metastasis and brain metastasis [7, 8], the prediction of drug efficacy [9], and the prediction of the risk of peripheral neuropathy caused by breast cancer treatment (paclitaxel) [10].

## Challenges of liquid biopsy with blood microRNA in breast cancer

Although all of these results are very important findings for the future of liquid biopsy for breast cancer, there are still significant hurdles to overcome in order to implement these new biomarkers into society as in vitro diagnostic agents for early detection of breast cancer. First, these laboratory-based findings must always be validated in a large-scale population study. A clinically meaningful diagnosis can be made only if, for example, after 10 years of continuous microRNA screening in a fixed population of tens of thousands of people, the mortality rate from breast cancer in that population has decreased. Currently, AMED and the National Cancer Center, with the support of the Japan Cancer Society, are conducting actual verification with the cooperation of medical institutions and others in five regions of Japan, including Okinawa and Hokkaido. In clinical trials, microRNAs in the blood are being measured and analyzed in comparison with the results of echocardiography for breast cancer to verify whether cancer can be detected. Also, even among those who have positive mammograms, the probability of actually having breast cancer is not so high. Another major focus is whether a system can be established to prevent unnecessary needle biopsies caused by these false positives by diagnosing blood microRNAs.

## Why are DNA, mRNA, and microRNAs in blood stable?

Why is the microRNA secreted by cancer cells stable when there must be many enzymes in the blood that degrade nucleic acids such as RNA? The reason for this was revealed in 2007. It was an epoch-making report that mRNAs and microRNAs are secreted and circulated in body fluids in the form of EVs [11]. The implication of this discovery is that microRNAs in these EVs are actively secreted by cancer cells as a means of survival in the patient's body and are delivered to target cells, where they specifically perform their functions, the very idea was that it was being used as a tool for information transmission between cells [12, 13]. In fact, this hypothesis has been proven in several empirical studies published since then [14], but in any case, reading the very number of microRNAs secreted by cancer cells leads to knowledge of the in vivo behavior of cancer cells, and consequently enables EV-based liquid biopsy for the diagnosis of cancer.

## Biological significance of EVs

The types of signaling molecules encapsulated by EVs are diverse. In addition to mRNAs and miRNAs, various non-coding RNAs, such as snoRNA, piRNA, and long non-coding RNA, have been detected. RNA alone is degraded in body fluids such as blood, especially because of the presence of RNase, but RNA encapsulated in EVs is protected from degradation and is stable even in body fluids. Proteins common to many cell-derived EVs include actin, tubulin, GAPDH, endosomal sorting proteins (ESCRT 0-III, Alix, Syntenin, Tsg101), heat shock proteins (HSP70, HSP90), and RAB families and annexin involved in membrane transport and fusion [15, 16]. Tetraspanins, transmembrane molecules, such as CD9, CD63, and CD81, are also localized on the EV surface. Thus, these proteins are often used as markers for EVs. The DNA in EVs that cancer cells are thought to secrete appears to be mostly double-stranded DNA of genomic origin. Recent findings have shown that DNA is not encapsulated within EVs as is the case with microRNAs, but rather resides on the surface of EVs [17], and it is highly likely that DNA is secreted through a pathway that is essentially different from that of secreted RNA molecules. Especially in the cancer microenvironment, where cancer cells survive by incorporating cancer-specific information into their own EVs and delivering this information to various surrounding cells in the tumor microenvironment. The EVs are then delivered to various surrounding cells, where they transmit the information. Thus, by controlling the surrounding cells, cancer cells can reign supreme in the microenvironment. This is precisely the reason why cancer cells make full use of EVs as a means of escaping immune cells that attack them.

## EVs in breast cancer metastasis

Metastasis is an extremely important factor that decreases the overall survival rate of breast cancer patients. EV miRNAs have been reported to play key roles in nearly every step of many biological processes in breast cancer development [18]. Many studies have demonstrated a dual role for EV miRNAs in breast cancer metastasis and related processes, successively elucidating several EV miRNAs and underlying mechanisms actively involved in metastasis, invasion, and migration. Furthermore, in brain metastasis of breast cancer, experimental data showed that miR-181c in breast cancer exosomes relaxes the binding of tight junctions of brain vascular endothelial cells, allowing breast cancer cells to easily cross the blood–brain barrier, and it was also reported that a large number of exosomes containing miR-181c can circulate in the blood of actual stage III and IV breast cancer patients with brain metastasis. It was also reported that a large number of exosomes containing miR-181c can circulate in the blood of stage III and IV breast cancer patients with brain metastases [19].

Bone metastasis of breast cancer is often osteolytic and well characterized by increased bone resorption. Bone destruction results in the release of several cytokines, such as interleukin-6 (IL-6), tumor necrosis alpha (TNFα), and transforming growth factor-beta 1 (TGF-β1), which promote tumor cell growth and invasion [20]. EVs released from breast cancer cells induce activation of osteoclast cells and destroy the bone remodeling [21].

In the lung metastasis of breast cancer, breast cancer-derived EVs play a role in pre-metastatic niche formation since breast cancer EVs contain multiple miRNAs, particularly miR-200b-3p, which is taken up by alveolar epithelial type II cells in the lung, thereby regulating the tumor microenvironment [22]. Thus, the breast cancer cell organotropic metastases to the lung are dependent on EV secretion and their transfer to the lung before the cancer cell invasion.

Presence of disseminated tumor cells (DTCs) in the bone marrow has been described as a surrogate of residual disease in patients with early breast cancer and DTCs are thought to be late recurrence of breast cancer patients. It is reported that EVs carrying microRNA-23b derived from bone marrow mesenchymal stem cells promote dormancy in metastatic breast cancer cells [23]. Thus, breast cancer cells survive for a long period. EV-mediated cancer metastasis is not limited to breast cancer. Some studies have reported the involvement of EVs in peritoneal metastasis of cancer. It has been shown that MMP1 mRNA in EVs secreted from ovarian cancer cells is taken up by peritoneal mesothelial cells and induces apoptosis, promoting peritoneal dissemination of cancer cells. Furthermore, MMP1 mRNA in EVs was identified to be present in ascites fluid, suggesting its usefulness as a biomarker to predict the risk of peritoneal dissemination [24].

## Significance of EVs in breast cancer stemness

Cancer stem cells are a population of undifferentiated cells that have the potential to replicate themselves and differentiate. Self-renewing cell populations cause tumor heterogeneity and promote metastasis, resistance to therapy, and tumor recurrence. Breast cancer stem cells (BCSCs), primarily in the quiescent G0 stage, have high DNA repair capacity and increase expression of ABC transporters, mediating drug resistance and tumorigenesis [25]. EV miRNAs play a pivotal role in the maintenance of cancer stem cells. EV miR-221/222 inhibits PTEN activity, which activates the Akt/NF-κB/COX-2 signaling pathway and promotes stemness-like traits in breast cancer cells [26]. EV miR-22 suppresses the TET/miR-200 axis by inhibiting TET (Ten Eleven Translocation DNA demethylase family) and increases stemness and EMT [27]. EV miRNAs may contribute to the maintenance of stemness by regulating the function and expression of genes associated with breast cancer stemness. Sox9 is an oncogenic transcription factor that induces transformation of mammary stem cells from differentiated breast epithelial cells, which is important for breast cancer development and malignancy [28], and EV miR-140 reduces the expression of this SOX2/SOX9 and works toward reducing stem cell populations [29]. Thus, microRNAs transported by exosomes play an important role in the cancer microenvironment of breast cancer for stem cell maintenance.

## Protein EV biomarkers for breast cancer

Proteins on the surface and inside EVs may also serve as cancer biomarkers for breast cancer. EVs exhibit characteristic protein expression profiles depending on their cellular origin, as shown, for example, by proteomic results available in the ExoCarta and EVPedia databases. These are reported to be proteins that interact with a wide variety of transmembrane and cytoplasmic signaling proteins [30, 31]. These EV membrane proteins, including tetraspanin CD9, metalloproteinase ADAM10, heat shock protein HSP70, and annexin-1, are known to be present in EVs secreted by breast cancer cells [32]. In addition, Wang et al. [33] recently showed that CD82-positive EVs levels in serum of breast cancer patients are significantly higher than in healthy controls, and that CD82 expression increases significantly with the progression of malignant breast cancer. Rapp et al. [34] reported that the epithelial cell adhesion molecules EpCAM and CD24 could be used as markers to specifically identify cancer-derived EVs in ascites and pleural effusions of breast and ovarian cancer patients. Breast and pancreatic cancers also contain members of the human epidermal growth factor receptor (HER) family [35]. In breast cancer cell lines that overexpress HER2, HER2-positive EVs modulate sensitivity to trastuzumab, which in turn modulates tumor aggressiveness by HER2. Kibria et al. [36] used flow cytometry to profile protein expression in EVs isolated from cell lines and blood of breast cancer patients and healthy individuals and observed a significant decrease in D47 expression in EVs of breast cancer patients compared to healthy controls. Certainly the reduction of CD47 in breast cancer patient EVs seems to make sense since its increased expression can prevent the innate immune system from recognizing cancer cells. All of these are promising EV biomarker candidates for breast cancer, but none have been put into practical use at this time.

## Conclusion

The potential of liquid biopsy in cancer care is great. In future, it is expected that early detection will not only reduce the cancer mortality rate, but also contribute significantly to reducing medical costs. However, the various possible challenges of public acceptance of these new diagnoses must also be fully considered. There are a variety of issues: the problem of overdiagnosis, the cost of medical checkups, how to explain the results, etc. on the part of medical professionals, how to smoothly link high-risk groups to diagnosis in a precise medical setting, competition with foreign countries, and so on. By overcoming these challenges one by one, we can provide a system that truly contributes to the early detection of cancer as a means of realizing Precision Medicine. In future, we hope that the biological activity of microRNAs and breast cancer-specific exosomes involved in breast cancer diagnosis and their molecular mechanisms will be clarified to better understand the changes in the pathogenesis of breast cancer.
